# High incidence of permanent pacemaker after Cox-maze IV and mitral valve surgery: a nationwide registry-based study

**DOI:** 10.1093/icvts/ivaf085

**Published:** 2025-04-04

**Authors:** Torbjörn Ivert, Gabriella Boano, Farkas Vanky, Fredrik Gadler, Anders Holmgren, Lena Jidéus, Birgitta Johansson, Göran Kennebäck, Shahab Nozohoor, Henrik Scherstén, Johan Sjögren, Anders Wickbom, Örjan Friberg, Anders Albåge

**Affiliations:** Department of Cardiothoracic Surgery, Karolinska University Hospital and Molecular Medicine and Surgery, Karolinska Institutet, Stockholm, Sweden; Department of Thoracic and Vascular Surgery, Unit of Cardiovascular Medicine, Linköping University, Linköping, Sweden; Department of Health, Medicine and Caring Sciences, Unit of Cardiovascular Medicine, Linköping University, Linköping, Sweden; Department of Thoracic and Vascular Surgery, Unit of Cardiovascular Medicine, Linköping University, Linköping, Sweden; Department of Health, Medicine and Caring Sciences, Unit of Cardiovascular Medicine, Linköping University, Linköping, Sweden; Department of Medicine, Karolinska Institutet, Solna, Stockholm, Sweden; Department of Cardiology, Karolinska University Hospital, Solna, Stockholm, Sweden; Heart Centre, University Hospital, Umeå, Sweden; Department of Public Health and Clinical Medicine, Umeå University, Umeå, Sweden; Department of Cardiothoracic Surgery and Anesthesiology, University Hospital, Uppsala, Sweden; Department of Surgical Sciences, University Hospital, Uppsala, Sweden; Department of Medicine, Geriatrics and Emergency Medicine, Sahlgrenska University Hospital/Östra, Gothenburg, Sweden; Department of Cardiology, Karolinska University Hospital, Karolinska Institutet, Stockholm, Sweden; Department of Clinical Sciences Lund, Lund University, Skåne University Hospital, Lund, Sweden; Department of Cardiothoracic Surgery, Lund University, Skåne University Hospital, Lund, Sweden; Department of Cardio-Thoracic Surgery, Sahlgrenska University Hospital, Gothenburg, Sweden; Department of Clinical Sciences Lund, Lund University, Skåne University Hospital, Lund, Sweden; Department of Cardiothoracic Surgery, Lund University, Skåne University Hospital, Lund, Sweden; Department of Cardiothoracic and Vascular Surgery, Faculty of Medicine and Health, Örebro University Hospital, Örebro, Sweden; Department of Cardiothoracic and Vascular Surgery, Faculty of Medicine and Health, Örebro University Hospital, Örebro, Sweden; Department of Cardiothoracic Surgery and Anesthesiology, University Hospital, Uppsala, Sweden; Department of Surgical Sciences, University Hospital, Uppsala, Sweden

**Keywords:** Cox-maze, mitral valve surgery, pacemaker

## Abstract

**OBJECTIVES:**

This study evaluated the long-term risk of permanent pacemaker implantation following Cox-maze IV (CMIV) and concurrent mitral valve surgery.

**METHODS:**

A retrospective, nationwide, registry-based analysis was conducted on postoperative permanent pacemaker implantation in 397 patients with symptomatic mitral valve insufficiency and atrial fibrillation who underwent CMIV and mitral valve surgery in Sweden between 2009 and 2017. They were compared to a registry group of 346 patients with atrial fibrillation who underwent mitral valve surgery without surgical ablation during 2014–2017. The follow-up ended on 30 September 2022.

**RESULTS:**

CMIV patients were on average 4 years younger and had lower surgical risk than registry patients. More CMIV patients underwent early (<30 days) pacemaker implantation (13.3% vs. 5.5%, *P *=* *0.002). CMIV patients had a doubled adjusted risk of requiring a pacemaker compared to registry patients after 8 years [HR 1.96, 95% CI 1.27–3.04]. In the CMIV group, 22% (95% CI 18–26%) had a pacemaker by 5 years, increasing to 27% (95% CI 22–31%) by 8 years, compared to 13% (95% CI 10–17%) at both time intervals in the registry group. Atrioventricular block II/III accounted for >60% of early pacemaker indications in both groups, and sinus node dysfunction was the indication for late pacemaker implantation in 48% in the CMIV group.

**CONCLUSIONS:**

Patients undergoing CMIV concomitant with mitral valve surgery have a higher rate of postoperative pacemaker implantation compared to patients with atrial fibrillation undergoing mitral valve surgery alone. Sinus node dysfunction was the main indication for late pacemaker among CMIV patients.

## GRAPHICAL ABSTRACT

**Figure:**
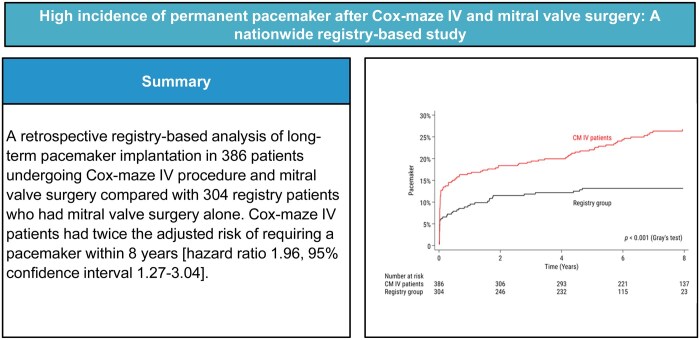

## INTRODUCTION

Atrial fibrillation is associated with an increased risk of stroke, heart failure and mortality [1–3]. Surgical treatment for atrial fibrillation using the Cox-maze procedure was introduced in the 1990s [4]. The original cut-and-sew technique (Cox-maze III) was later replaced by Cox-maze IV (CMIV), where atrial lesions are treated more quickly and efficiently using cryoablation or bipolar radiofrequency ablation [5, 6]. Freedom from atrial fibrillation has been reported in more than 90% of treated patients [7–9].

The need for a permanent pacemaker after modifications to the initial ‘cut-and-sew’ technique by Cox *et al.* decreased from 56% to 25% in patients followed for at least 3 months [10]. Bradyarrhythmia requiring an early permanent pacemaker after CMIV procedures has been reported in 6–25% of cases [11–19]. Pecha *et al.* reported a pacemaker rate of 13.6% within 30 days following biatrial ablation of lesions [20]. DeRose *et al.* recorded a 25% rate of permanent pacemaker implantation during the initial hospitalization following combined mitral valve surgery and biatrial maze lesions [17]. Robertson *et al.* reported a 15% rate of permanent pacemaker implantation 1 year after concomitant CMIV surgery [15]. However, there are no reports on the long-term risk of permanent pacemaker placement following concomitant CMIV and mitral valve surgery.

This study aimed to analyse the long-term need for permanent pacemaker placement after CMIV in patients with mitral insufficiency and atrial fibrillation who underwent concurrent mitral valve surgery. The rate of pacemaker implantation was compared to a registry group of patients with atrial fibrillation who underwent surgery for mitral valve insufficiency between 2014 and 2017 but did not undergo CMIV or other surgical ablation procedures.

## PATIENTS AND METHODS

### Patients

In a nationwide retrospective observational study, we identified all 443 patients over the age of 20 with symptomatic mitral valve disease and atrial fibrillation who had undergone elective combined biatrial Cox-maze procedure and mitral valve surgery (repair or replacement) with or without tricuspid valve surgery (repair or replacement) at seven surgical units in Sweden between 2009 and 2017. Patients undergoing other concomitant cardiac procedures were excluded, including those with other lesion sets for surgical ablation besides CMIV. To obtain stronger homogeneity, we excluded patients with mitral stenosis and concomitant ‘cut-and-sew’ Cox-maze III, leaving a study group of 397 patients for the analysis (Table 1, Supplementary Fig. S1). All hospital records covering the perioperative period were reviewed for patients in the study group. All patients who were reported with a pacemaker inserted before the operation were subsequently excluded from all analyses of rate of postoperative pacemaker. All data were anonymized before analysis and obtained from the Swedish National Board of Health and Welfare on 2 November 2022. Informed consent was not required for the analysis of national register data. This study complied with the principles of the Declaration of Helsinki and was approved by the Regional Ethical Review Board of Stockholm (Dnrs. 2018/608-31 and 2024-01229-02).

**Table 1:** Baseline characteristics in patients who had Cox-maze IV for atrial fibrillation and in registry patients without Cox-maze IV

Variable	Cox-maze IV		Registry		*P*-value
	(*n *=* *397)		(*n *=* *346)		
	Mean	SD	Mean	SD	
Age (years)	65.7	9.0	69.5	10.1	<0.001
Body mass index (kg/m^2^)	25.5	4.1	25.9	4.6	0.13
Creatinine (µmol/L)	91.3	22.7	96.2	33.3	0.06
	Median	IQR	Median	IQR	
EuroSCORE II	1.8	0.1–3.8	3.6	1.9–6.9	<0.001
	*n*	%	*n*	%	
Females	93	23.4	113	32.7	0.007
Hypertension	123	31.0	79	22.8	0.02
Diabetes mellitus	14	3.5	34	9.8	<0.001
Paroxysmal atrial fibrillation	115	29.0	145	41.9	<0.001
Non-paroxysmal atrial fibrillation	282	71.0	201	58.1	<0.001
Previous					
Pacemaker	11	2.7	42	12.1	<0.001
Stroke/transient ischaemic attack	30	7.6	44	12.7	0.03
Cardiac surgery	16	4.0	69	19.9	<0.001
New York Heart Association class					
I	21	5.3	9	2.6	0.09
II	144	36.3	64	18.5	<0.001
III	211	53.1	193	55.8	0.52
IV	6	1.5	78	22.5	<0.001
Left ventricular ejection fraction					
>50%	235	59.2	205	59.2	0.95
31–50%	137	34.5	131	37.9	0.38
21–30%	5	1.3	8	2.3	0.42
<21%			2	0.6	

BMI: body mass index; IQR: interquartile range; EuroSCORE: European System for Cardiac Operative Risk Evaluation Score; NYHA: New York Heart Association; LVEF: left ventricular ejection fraction.

In the SWEDEHEART (Swedish Web-system for Enhancement and Development of Evidence-based Care in Heart Disease Evaluated According to Recommended Therapies) register, we identified a registry group of 367 patients with preoperative atrial fibrillation and mitral valve disease who had elective mitral valve surgery between 2014 and 2017 and did not undergo a CMIV procedure or any other surgical ablation. We excluded patients with mitral stenosis, leaving 346 patients in the registry group (Table 1, Supplementary Fig. S1). Valid information regarding preoperative or previous atrial fibrillation was unavailable in the SWEDEHEART register prior to 2014.

### Methods

Each resident in Sweden is assigned a unique personal identity number, which for the follow-up of our patients in both groups was linked to the National Patient Register, Cause of Death Register and Swedish ICD and Pacemaker Registry. The Swedish National Board of Health and Welfare established record linkages. The Cause of Death Register was used to confirm vital status and determine dates of death, and the follow-up period concluded on 30 September 2022.

The surgical technique for the CMIV procedure has been described in detail and emphasizes strict adherence to the lesion pattern and the biatrial approach [5, 21, 22]. The CMIV lesions in the atria were created using either cryoablation or a nitrous oxide-based cryoablation probe in 360 patients (90.7%), or bipolar radiofrequency ablation in 37 patients (9.3%) [23]. Left atrial appendage closure was performed according to the surgeon’s discretion, with closure achieved in 203 patients undergoing CMIV (51.1%). In 69 patients (34.0%), a closing device was used; in the remaining cases, the ostium of the appendage was sutured from inside the left atrium. The patients continued anticoagulation treatment after the CMIV procedure, typically with direct oral anticoagulants.

### Definitions

All baseline patient characteristics and diagnoses were based on data reported from contributing centres and the 10th revision of the International Classification of Diseases (ICD-10) codes in the registers (Supplementary Table S1) Atrial fibrillation was defined as any episode ≥30 s recorded on continuous telemetry or a 12-lead electrocardiogram. The Nordic Medico-Statistical Committee Classification of surgical procedure codes FPE 00 to FPE 30 was used to identify the date of pacemaker implantation. Body mass index (BMI) was calculated as weight in kilograms divided by the square of height in metres. The preoperative surgical risk was assessed using the European System for Cardiac Operative Risk Evaluation Score II (EuroSCORE II) [24].

### Statistical methods

Data are presented as arithmetic means with standard deviations (SD) or, in cases of non-Gaussian distributions, as medians with interquartile range (IQR). Student’s *t* test or Mann–Whitney *U* test was applied for continuous variables, and the Yates-corrected chi-squared test was used to compare proportions of categorical variables between the two groups. The Kaplan–Meier method was used to calculate cumulative survival, and the Cox–Mantel test was employed to compare the two groups [25]. Cox proportional hazard regression was used to estimate the crude and adjusted hazard ratios for pacemaker implantation. The Aalen-Johansen estimator was used to obtain the cumulative incidence function for pacemaker implantation while accounting for the competing risk of death. We estimated crude and adjusted subdistribution hazard ratios for pacemaker implantation using competing-risks regression models according to the method of Fine and Gray. A *P*-value <0.05 was considered statistically significant. Calculations were performed using STATISTICA 13 (Stat Soft, Dell) and Stata 18.5 (Stata Corp LLC, College Station, TX, USA).

## RESULTS

CMIV patients were, on average, 4 years younger and had a lower EuroSCORE than those in the registry group (Table 1). Female sex, diabetes mellitus, paroxysmal atrial fibrillation, prior permanent pacemaker use, history of stroke or cardiac surgery, and New York Heart Association (NYHA) functional class IV were less common, whereas hypertension was more common among CMIV patients compared to registry patients. Cardiopulmonary bypass and aortic cross-clamp times were longer, and mitral and tricuspid valve repairs were performed more frequently, whereas postoperative renal replacement therapy was required less often in the CMIV group compared to the registry group (Table 2).

**Table 2:** Perioperative data in patients who had Cox-maze IV for atrial fibrillation and registry patients without Cox-maze IV

Variable	Cox-maze IV (*n *=* *397)		Registry (*n *=* *346)		*P*-value
	Mean	SD	Mean	SD	
Cardiopulmonary bypass (min)	119.2	36.0	91.8	32.4	<0.001
Aortic cross-clamp time (min)	168.2	50.4	137.4	57.9	<0.001
Valve procedure	*n*	%	*n*	%	
Mitral valve repair	340	85.6	183	52.9	<0.001
Mechanical valve	45	11.3	163	47.1	<0.001
Bioprosthetic valve	12	3.0	0	0.0	
Tricuspid valve repair	174	43.8	103	29.8	0.001
Tricuspid valve replacement	4	1.0	0	–	
Early postoperative complications					
Reoperation for bleeding	25	6.3	17	4.9	0.51
Stroke	4	1.0	4	1.2	0.88
Renal replacement therapy	7	1.8	23	6.6	0.001
Mediastinitis	4	1.0	2	0.6	0.82

### Follow-up

**Figure 1:**
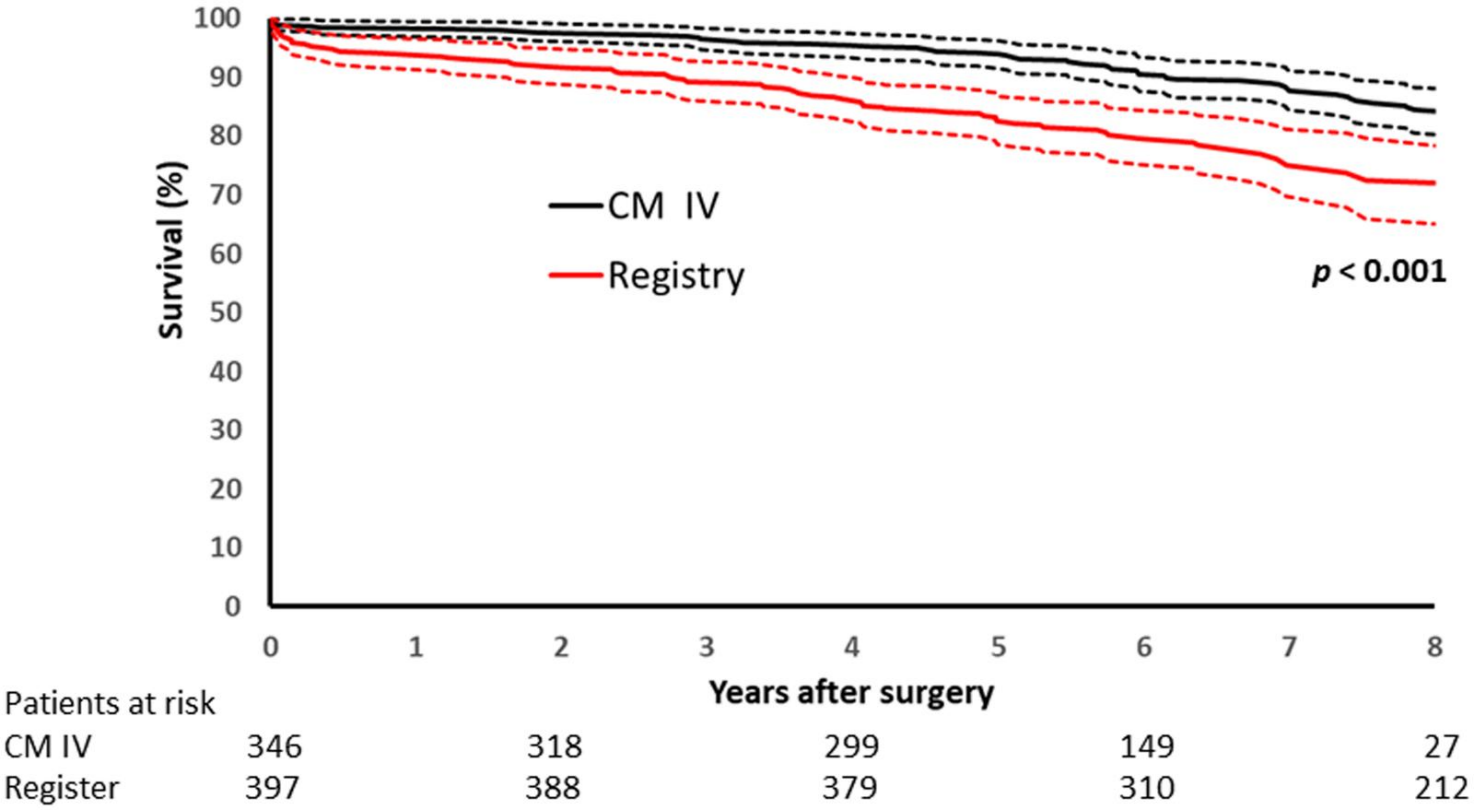
Crude survival in all Cox-maze IV (CMIV) and registry patients with 95% confidence intervals.

There were four early deaths (1%) and 74 late deaths in the CMIV group compared to 10 early deaths (2.9%) and 65 late deaths in the registry group (Table 3). Congestive heart failure was the most common cause of mortality overall. The median follow-up time was 2.5 years longer in the CMIV group (8.6 years, range 4.8–13.4 years) than in the registry group (6.1 years, range 4.8–8.7 years) (*P *<* *0.001). Crude survival at 5 and 8 years was higher in the CMIV group (94% and 85%, respectively) compared to the registry group (82% and 72%, respectively) (Fig. 1). Most pacemakers were implanted during the first year after the operation in CMIV patients (Supplementary Fig. S2). The median age at pacemaker implantation was 69 years (IQR 62–74) (Supplementary Fig. S3). Among the 690 patients who did not have pacemaker before the operation, 103 permanent pacemakers were inserted in the CMIV group and 40 in registry patients (Table 3). The rate of pacemaker was 20% (99/495) after mitral valve repair vs. 22% (44/195) after mitral valve replacement (*P *=* *0.52). One of four patients (25%) had pacemaker after tricuspid valve replacement vs. 29% (74/259) after tricuspid valve repair (*P* = 0.69). A pacemaker was inserted in 75 of 263 patients (29%) who had tricuspid valve repair or replacement and in 68 of 427 patients (16%) who did not have tricuspid valve surgery (*P* = 0.01). We found no evidence of effect modification of tricuspid valve surgery on the risk of postoperative permanent pacemaker implantation (*P* for interaction = 0.62).

**Table 3:** Outcomes in the Cox-maze IV and registry patients

	Cox-maze IV (*n *=* *397)		Registry (*n* = 346)		*P*-value
	*n*	%	*n*	%	
Deaths					
≤30 days	4	(1.0%)	10	(2.9%)	0.11
Late	74	(18.3%)	65	(18.8%)	0.97
Total	78	(19.6%)	75	(21.7%)	0.55
Cause of deaths					
Stroke	4		1		
ICH	3		6		
CHF	45		40		
Myocardial infarction	8		15		
Malignancy	18		13		
No preoperative pacemaker	386		304		
Postoperative pacemaker	*n*	%^a^	*n*	%^a^	
<30 days	49	12.7	19	6.3	0.007
≥30 days to <5 years	36	9.3	21	6.9	0.31
≥5 years to <8 years	15	3.9			
≥8 years	3	0.8			
Total after surgery	103	25.9	40	11.6	

ICH: intracranial haemorrage; CHF: congestive heart failure.

a Percentage of patients who did not have pacemaker before the operation.

The preoperative median duration of atrial fibrillation was 6 months (IQR 1–12) in patients who required postoperative pacemaker vs. 4 months (IQR 1–12) in those without pacemaker (*P* = 0.03). Among the 690 patients without preoperative pacemaker, suture closure of the atrial appendage was associated with 25% (50/197) postoperative pacemaker, 7% (13/197) postoperative bleeding and 3% (5/197) postoperative dialysis. The corresponding figures for a device to close the left atrial appendage were 11% (7/65) pacemaker, 5% (3/65) postoperative bleeding and 8% (5/65) postoperative dialysis. The rate of pacemaker implantation during the first postoperative month did not differ between the cryoablation (45/360, 12.54%) and the radiofrequency ablation group (4/37, 10.8%) (*P *=* *0.83).

**Figure 2:**
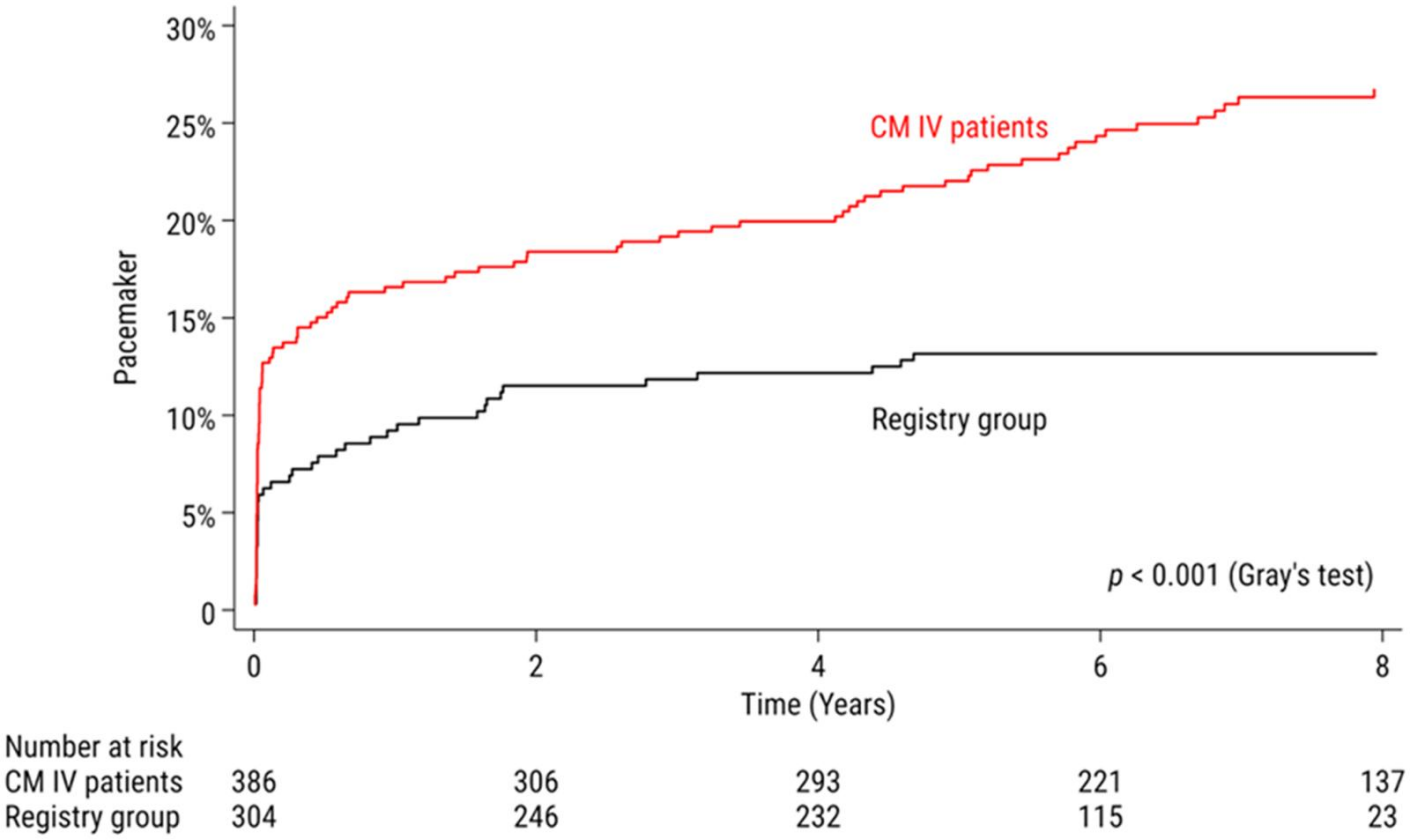
Cumulative incidence of pacemaker implantation following Cox-maze IV and in registry patients. Patients with pacemakers implanted before the operation are excluded.

Eight years after the operation, CMIV patients had twice the adjusted risk of requiring a pacemaker compared to registry patients (Table 4). In the CMIV group, 22% (95% confidence interval [CI] 18–26%) had a pacemaker at 5 years, increasing to 27% (95% CI 22–31%) at 8 years, compared to 13% (95% CI 10–17%) at both 5 and 8 years in the registry group (Fig. 2).

**Table 4:** Crude and adjusted risk of pacemaker implantation after Cox-maze IV

Model	Subdistribution hazard ratio (95% CI)	*P*-value
Crude	2.03 (1.41–2.92)	<0.001
Adjusted for age and sex	2.21 (1.52–3.24)	<0.001
Adjusted for EuroSCORE II	2.43 (1.60–3.71)	<0.001
Adjusted for EuroSCORE II and TVR	1.99 (1.29–3.06)	0.002
Adjusted for EuroSCORE II, TVR and non-paroxysmal atrial fibrillation	1.96 (1.27–3.04)	0.003

The analysis was conducted using competing-risks regression models according to the method of Fine and Gray and accounted for the competing risk of death in 690 patients without preoperative pacemaker and the reference group was registry patients.

CI: confidence interval, EuroSCORE: European System for Cardiac Operative Risk Evaluation Score; TVR: tricuspid valve repair/replacement.

Atrioventricular blocks II and III accounted for over 60% of the indications for pacemaker implantation during the first month in both groups. Sinus node dysfunction was an indication for postoperative pacemaker in 22% of CMIV patients vs. 5% of registry patients (*P *=* *0.49) during the first 30 days and after the first 30 days in 48% of CMIV patients and 14% of registry patients (*P *=* *0.01) (Table 5). In addition, a pacemaker was implanted due to symptomatic bradycardia in 18 patients in each group. All patients were symptomatic before pacemaker implantation. In total, 13 patients experienced syncope, and 7 were resuscitated after cardiac arrest. Characteristics of the CMIV and registry patients with and without pacemakers are listed in Supplementary Tables S2 and S3. CMIV patients with pacemakers underwent mitral valve prostheses and tricuspid valve repair more frequently than those without a pacemaker.

**Table 5:** Postoperative pacemaker (PM) indications and symptoms

ECG findings	Cox-maze IV (*n* = 386)					Registry (*n* = 304)				
	PM	Atrioventricular block		Sinus node dysfunction		PM	Atrioventricular block		Sinus node dysfunction	
	*n*	*n*	%	*n*	%	*n*	*n*	%	*n*	%
Postoperative <30 days	49	32	65.3	11	22.4	19	12	63.2	1	5.3
Postoperative ≥30 days	54	14	25.9	26	48.1	21	6	28.6	3	14.3
Total pacemakers	103	48	46.6	37	35.9	40	18	45.0	4	10.0

Medical history		Syncope		Cardiac arrest			Syncope		Cardiac arrest	
	*n*	*n*	%	*n*	%	*n*	*n*	%	*n*	%
Postoperative <30 days	49	2	4.1	2	4.1	19	0	0	1	5.3
Postoperative ≥30 days	54	10	18.5	3	5.6.	21	1	4.7	1	4.8
Total pacemakers	103	12	11.7	5	4.9	40	1	2.5	2	5.0

## DISCUSSION

This is a nationwide study including all concomitant Cox-maze and mitral valve procedures in Sweden performed between 2009 and 2017. By linking a unique personal identity number to national health registers, we were able to trace every death and hospitalization, including the date and indication for all pacemaker implantations before and after the operation. Our national health registers are of high quality, and data regarding heart surgery in the SWEDEHEART registry have been audited and validated [26]. None of the patients were lost to follow-up. Pacemaker data were obtained from the National Patient Register and the Swedish ICD and Pacemaker Registry.

Patients included in the registry group had preoperative atrial fibrillation and mitral valve surgery but did not undergo concomitant arrhythmia surgery. Possible reasons for this exclusion may have included asymptomatic atrial fibrillation, advanced age, previous heart surgery, or a planned Cox-maze procedure that was not performed due to intraoperative difficulties or other factors. The two groups shared common features of atrial fibrillation and significant mitral valve insufficiency but differed in that registry patients were older and more frequently had diabetes, prior pacemaker use and lower survival rates compared to CMIV patients. After multivariate adjustment, pacemaker implantation up to 8 years after the operation was performed twice as often in CMIV patients compared to registry patients.

The need for a pacemaker increases with age [15]. The median age at pacemaker implantation was 69 years. Indications for pacemaker implantation include bradycardia, sinus node dysfunction and atrioventricular block. Mitral valve prostheses and tricuspid valve repair may increase the risk of an atrioventricular block [27]. Electrocardiography was performed, and symptoms were documented at the time of pacemaker implantation. In our CMIV group, sinus node dysfunction was an indication for pacemaker implantation in 22% of patients during the first postoperative month and in approximately half of the late-implanted pacemakers. Sinus node dysfunction was present in 79% of the patients requiring a pacemaker after CMIV, as reported by Robertson *et al.* [15]. There may be a dysfunctional sinoatrial node before the operation, and the Cox-maze procedure may unmask the sick sinus node [28]. Furthermore, the absence of atrial contraction after Cox-maze procedures has been associated with an increased risk of permanent pacemaker implantation [29]. Low-amplitude atrial fibrillation is reported as one cause of the need for a pacemaker after the Cox-maze procedure, while angiotensin-converting enzyme inhibitors and angiotensin receptor blockers have been shown to reduce the need for pacemakers [14].

Reports in the literature indicate that right atrial ablation lesions, as used in CMIV, increase the rate of postoperative permanent pacemaker implantation [11, 17, 20]. In a meta-analysis, the maze procedure was associated with the highest rates of postoperative sinus node dysfunction and permanent pacemaker implantation [30]. Lesions in the right atrium can lead to conduction disorders in the tissue surrounding the sinus node. Microwave energy has also been reported to correlate with an increased risk of permanent pacemaker implantation [11]. In our analysis, the need for a pacemaker did not correlate with the type of energy modality used. The need for a permanent pacemaker after surgical ablation for atrial fibrillation has been associated with higher 3-month and 1-year mortality [11, 17].

### Limitations

The limitations inherent to this multicentre nationwide retrospective observational study of patients with mitral insufficiency who underwent the Cox-maze procedure include the potential for incomplete and inaccurate data, unknown confounders and variations in clinical practices across the country. We did not have any measure of left atrial enlargement that may have influenced outcome of the CMIV procedure. The threshold for pacemaker requirements may vary over time and between centres. Our analyses did not provide evidence that pre-existing sinus or atrioventricular node dysfunction or right atrial lesions were the primary causes of the need for a postoperative pacemaker. There were significant differences between the two patient groups. We believe that our conclusion of a higher pacemaker rate in Cox-maze patients is valid, based on the calculated multivariable-adjusted risk. However, we do not have detailed information regarding long-term postoperative arrhythmia or compliance with medication.

## CONCLUSION

The CMIV procedure combined with mitral valve surgery can be performed with low perioperative risk. However, the CMIV procedure combined with mitral valve surgery was associated with a higher rate of postoperative pacemaker use compared to mitral valve surgery without CMIV in patients with atrial fibrillation. Sinus node dysfunction was the main indication for late pacemaker among CMIV patients. There was no effect modification of tricuspid valve surgery on the risk of postoperative pacemaker implantation.

## Supplementary Material

ivaf085_Supplementary_Data

## Data Availability

Anonymized data underlying this article will be available on request to the corresponding author.

## ABBREVIATIONS

CI: Confidence interval
CMIV: Cox-maze IV
IQR: Interquartile range
