# Supplementary material for: High incidence of permanent pacemaker after Cox-maze IV and mitral valve surgery: a nationwide registry-based study
Source: Interdiscip Cardiovasc Thorac Surg. 2025 Apr 4;40(4):ivaf085. doi: 10.1093/icvts/ivaf085 (PMC12005902; doi:10.1093/icvts/ivaf085)
Supplement: ivaf085_Supplementary_Data [file ivaf085_supplementary_data.zip › Supplementary Tables S1 to S3.docx]

**Supplementary Tables S1 to S3**

**S1 Table:** International Classification of Diseases (ICD-10) codes

| Hypertension | I10-I15.9 |
| --- | --- |
| Diabetes | E10-E14.9 |
| Myocardial infarction | I21–I21.9 |
| Heart failure | I50–I50.9, I42-I43.9, I25.5, I11.0 |
| Cerebral bleeding | I60.0–I62.9 |
| Ischemic stroke | I63 |
| Atrial fibrillation | I48 |

**S2 Table:** Characteristics in Cox-maze IV patients in relation to all implanted pacemakers

|  | Pacemaker  (*n*=114) | | No pacemaker  (*n*=283) | | *p* Value |
| --- | --- | --- | --- | --- | --- |
| *Variable* | *Mean* | *SD* | *Mean* | *SD* |  |
| Age (yrs) | 67.0 | 9.0 | 65.1 | 9.0 | 0.05 |
| BMI (kg/m^2^) | 25.2 | 3.9 | 25.7 | 4.2 | 0.34 |
| Creatinine (µmol/L) | 91.5 | 27.9 | 91.2 | 27.9 | 0.92 |
|  | *Median* | *IQR* | *Median* | *IQR* |  |
| EuroSCORE II | 2.9 | 0.5-5.0 | 1.5 | 0.1-2.9 | 0.001 |
|  | *n* | *%* | *n* | *%* |  |
| Females | 35 | 30.7 | 58 | 20.5 | 0.04 |
| Hypertension | 41 | 36.0 | 82 | 29.0 | 0.21 |
| Diabetes | 5 | 4.4 | 9 | 3.2 | 0.77 |
| Paroxysmal AF | 29 | 25.4 | 86 | 30.4 | 0.39 |
| Non-paroxysmal AF | 85 | 74.6 | 197 | 69.6 | 0.39 |
| Previous: |  |  |  |  |  |
| Stroke /TIA | 9 | 7.9 | 25 | 8.8 | 0.92 |
| Cardiac surgery | 7 | 6.1 | 9 | 3.2 | 0.28 |
| NYHA-functional class |  |  |  |  |  |
| I | 6 | 5.3 | 15 | 6.3 |  |
| II | 36 | 31.6 | 108 | 38.2 | 0.26 |
| III | 67 | 58.8 | 144 | 50.9 |  |
| IV | 2 | 1.8 | 4 | 1.4 |  |
| LVEF |  |  |  |  |  |
| >50% | 56 | 49.1 | 178 | 63.3 |  |
| 31-50% | 49 | 43.0 | 88 | 31.1 | 0.03 |
| 21-30% | 3 | 2.6 | 2 | 0.7 |  |
| < 20% |  |  |  |  |  |
| Mitral valve repair | 89 | 78.1 | 25 | 88.7 | 0.01 |
| Mitral valve replacement | 25 | 24.2 | 32 | 11.3 | 0.01 |
| Tricuspid valve repair | 62 | 54.4 | 112 | 39.6 | 0.01 |
| Tricuspid replacement | 1 | 0.9 | 3 | 1.1 | 0.69 |

BMI: body mass index; IQR: interquartile range; AF: atrial fibrillation; EuroSCORE: European System for Cardiac Operative Risk Evaluation; NYHA: New York Heart Association classification; LV: Left Ventricular Ejection Fraction.

**S3 Table.** Characteristics in Registry patients in relation to all implanted pacemakers

|  | Pacemaker  (*n*=82) | | No pacemaker  (*n*=264) | | *p* Value |
| --- | --- | --- | --- | --- | --- |
| *Variable* | *Mean* | *SD* | *Mean* | *SD* |  |
| Age (yrs) | 69.4 | 10.9 | 69.5 | 9.9 | 0.91 |
| BMI (kg/m^2^) | 26.0 | 4.7 | 25.8 | 4.6 | 0.75 |
| Creatinine (µmol/L) | 97.7 | 44.6 | 95.7 | 28.7 | 0.78 |
|  | *Median* | *IQR* | *Median* | *IQR* |  |
| EuroSCORE II | 3.6 | 2.2-8.4 | 3.5 | 1.8-5.9 | 0.42 |
|  | *n* | *%* | *n* | *%* |  |
| Females | 28 | 34.1 | 85 | 32.2 | 0.84 |
| Hypertension | 24 | 29.3 | 55 | 20.8 | 0.15 |
| Diabetes | 9 | 11.0 | 25 | 9.5 | 0.85 |
| Paroxysmal AF | 27 | 32.9 | 118 | 44.7 | 0.08 |
| Non-paroxysmal AF | 55 | 67.1 | 146 | 55.3 | 0.08 |
| Previous: |  |  |  |  |  |
| Stroke /TIA | 15 | 18.3 | 29 | 11.1 | 0.12 |
| Cardiac surgery | 22 | 26.8 | 47 | 17.8 | 0.10 |
| NYHA-functional class |  |  |  |  |  |
| I | 2 | 2.4 | 22 | 4.0 |  |
| II | 10 | 12.2 | 162 | 29.6 | <0.001 |
| III | 49 | 59.8 | 288 | 52.7 |  |
| IV | 21 | 25.6 | 61 | 11.2 |  |
| LVEF |  |  |  |  |  |
| >50% | 44 | 53.7 | 161 | 61.0 |  |
| 1-50% | 35 | 42.7 | 96 | 36.4 | 0.37 |
| 21-30% | 2 | 2.4 | 6 | 2.7 |  |
| < 20% | 1 | 1.2 | 1 | 0.4 |  |
| Mitral valve repair | 38 | 46.3 | 145 | 54.9 | 0.22 |
| Mitral valve replacement | 44 | 53.7 | 119 | 45.1 | 0.22 |
| Tricuspid valve repair | 30 | 36.6 | 73 | 27.7 | 0.16 |
| Tricuspid replacement | 0 |  | 3 | 1.1 |  |

BMI: body mass index; IQR: interquartile range; AF: atrial fibrillation; EuroSCORE: European System for Cardiac Operative Risk Evaluation; NYHA: New York Heart Association classification; LV: Left Ventricular Ejection Fraction.
