# Supplementary material for: High incidence of permanent pacemaker after Cox-maze IV and mitral valve surgery: a nationwide registry-based study
Source: Interdiscip Cardiovasc Thorac Surg. 2025 Apr 4;40(4):ivaf085. doi: 10.1093/icvts/ivaf085 (PMC12005902; doi:10.1093/icvts/ivaf085)
Supplement: ivaf085_Supplementary_Data [file ivaf085_supplementary_data.zip › Supplementary Figures S1 to S3.docx]

**Supplementary Figures S1 to S3**

**
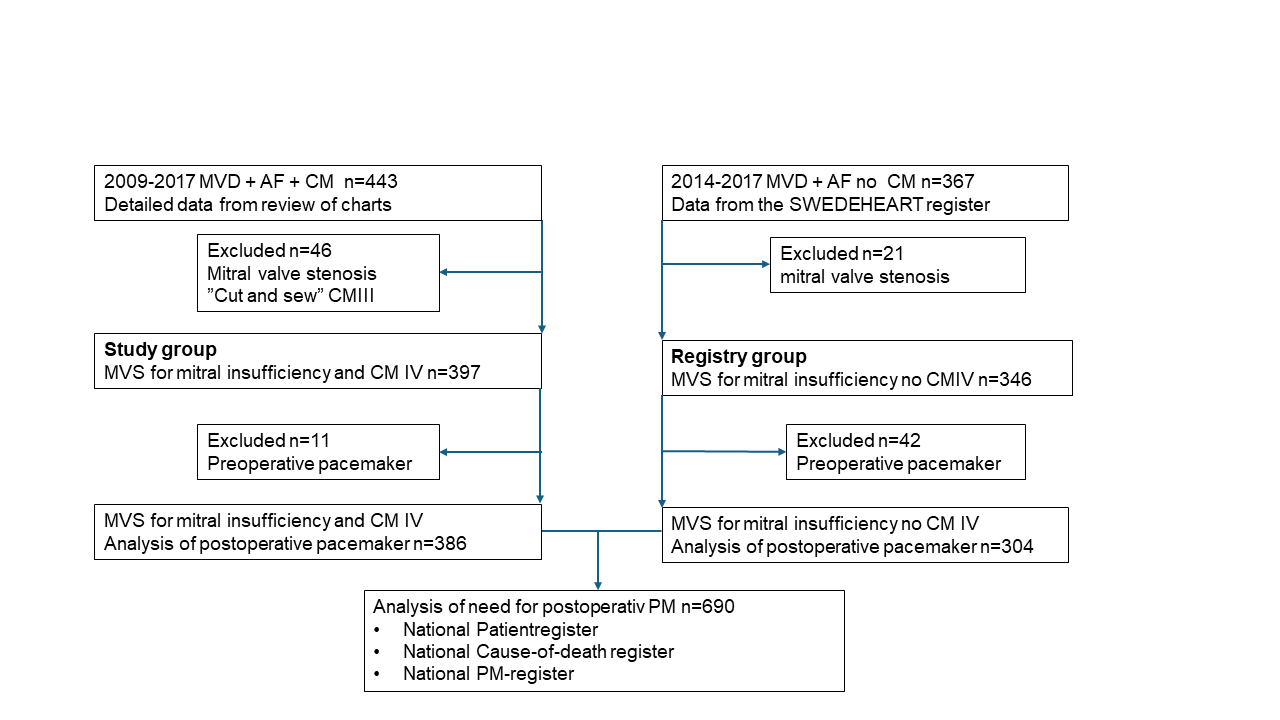
**

**Supplementary Figure S1.** Flowchart of all patients in Sweden with atrial fibrillations (AF) undergoing mitral valve surgery (MVS) for mitral valve disease (MVD) and concomitant Cox-maze (CM) procedure during 2009-2017 and registry patients who had no surgical ablation.

**
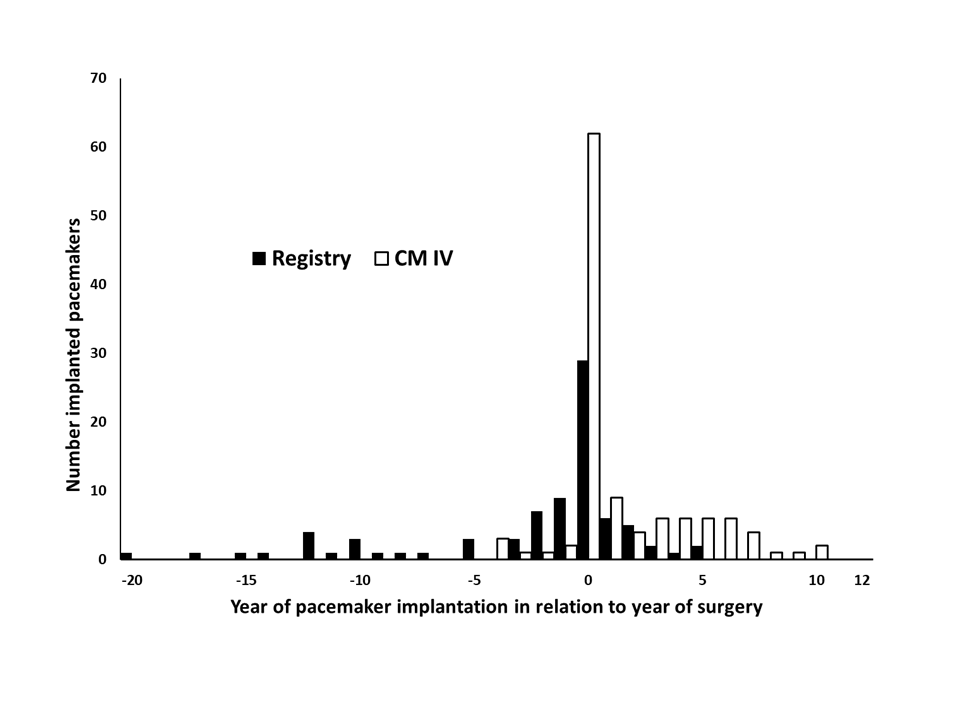
Supplementary Figure S2.** Year of all implanted pacemakers in Cox-maze IV (CM IV) and registry patients in relation to year of surgery


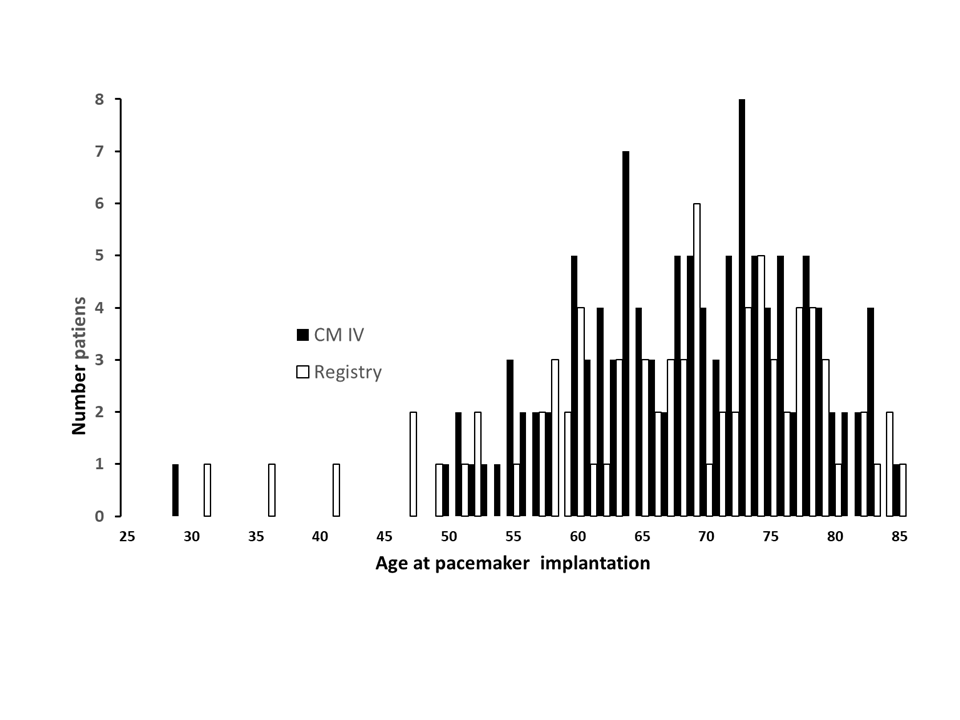


**Supplementary Figure S3.** Age at all pacemaker implantations in Cox-maze IV (CM IV) and registry patients
